# Genomic Analysis of Storage Protein Deficiency in Genetically Related Lines of Common Bean (*Phaseolus vulgaris*)

**DOI:** 10.3389/fpls.2016.00389

**Published:** 2016-03-31

**Authors:** Sudhakar Pandurangan, Marwan Diapari, Fuqiang Yin, Seth Munholland, Gregory E. Perry, B. Patrick Chapman, Shangzhi Huang, Francesca Sparvoli, Roberto Bollini, William L. Crosby, Karl P. Pauls, Frédéric Marsolais

**Affiliations:** ^1^Department of Biology, University of Western Ontario, LondonON, Canada; ^2^Genomics and Biotechnology, London Research and Development Centre, Agriculture and Agri-Food Canada, LondonON, Canada; ^3^Department of Bioscience and Biotechnology, School of Life Sciences, Sun Yat-sen UniversityGuangzhou, China; ^4^Department of Biological Sciences, University of Windsor, WindsorON, Canada; ^5^Department of Plant Agriculture, University of Guelph, GuelphON, Canada; ^6^Institute of Agricultural Biology and Biotechnology, National Research CouncilMilan, Italy

**Keywords:** genome sequencing, introgression, deletion, lectin, phaseolin, common bean, *Phaseolus vulgaris*

## Abstract

A series of genetically related lines of common bean (*Phaseolus vulgaris* L.) integrate a progressive deficiency in major storage proteins, the 7S globulin phaseolin and lectins. SARC1 integrates a lectin-like protein, arcelin-1 from a wild common bean accession. SMARC1N-PN1 is deficient in major lectins, including erythroagglutinating phytohemagglutinin (PHA-E) but not α-amylase inhibitor, and incorporates also a deficiency in phaseolin. SMARC1-PN1 is intermediate and shares the phaseolin deficiency. Sanilac is the parental background. To understand the genomic basis for variations in protein profiles previously determined by proteomics, the genotypes were submitted to short-fragment genome sequencing using an Illumina HiSeq 2000/2500 platform. Reads were aligned to reference sequences and subjected to *de novo* assembly. The results of the analyses identified polymorphisms responsible for the lack of specific storage proteins, as well as those associated with large differences in storage protein expression. SMARC1N-PN1 lacks the lectin genes *pha-E* and *lec4-B17*, and has the pseudogene *pdlec1* in place of the functional *pha-L* gene. While the α-phaseolin gene appears absent, an approximately 20-fold decrease in β-phaseolin accumulation is associated with a single nucleotide polymorphism converting a G-box to an ACGT motif in the proximal promoter. Among residual lectins compensating for storage protein deficiency, mannose lectin FRIL and α-amylase inhibitor 1 genes are uniquely present in SMARC1N-PN1. An approximately 50-fold increase in α-amylase inhibitor like protein accumulation is associated with multiple polymorphisms introducing up to eight potential positive *cis*-regulatory elements in the proximal promoter specific to SMARC1N-PN1. An approximately 7-fold increase in accumulation of 11S globulin legumin is not associated with variation in proximal promoter sequence, suggesting that the identity of individual proteins involved in proteome rebalancing might also be determined at the translational level.

## Introduction

Storage protein deficiency in crops is compensated through a mechanism of proteome rebalancing, whereby seed protein concentration is maintained at its normal level (Herman, 2014; Wu and Messing, 2014). This property has been used to express foreign recombinant protein (Schmidt and Herman, 2008; Lin et al., 2013; Hegedus et al., 2014) and for protein quality improvement (Kita et al., 2009; Wu et al., 2013; Kim et al., 2014). In soybean, because seed protein composition influences tofu quality, research has been performed to identify genetic variants for major seed proteins (Liu et al., 2006; Hayashi et al., 2009; Tsubokura et al., 2012; Kim et al., 2013; Wang et al., 2014) and to mobilize this genetic variation into cultivated varieties through marker-assisted selection (Jegadeesan et al., 2012; Song et al., 2014).

Common bean (dry bean, *Phaseolus vulgaris*) is the most important food legume for direct human consumption. A set of genetically related lines integrating a progressive deficiency in major storage proteins has been described (Osborn et al., 2003). The 7S globulin and major lectins are encoded at two unique loci. The major lectin or arcelin-phytohemagglutinin-α-amylase inhibitor (APA) locus in SARC1 is derived from the wild accession G12882 and includes the insecticidal lectin arcelin-1. SMARC1-PN1 and SMARC1N-PN1 integrate a deficiency in phaseolin introduced from a *Phaseolus coccineus* accession. SMARC1N-PN1 further integrates a lectin deficiency from the cultivar Great Northern 1140. The three lines share a common genetic background from the cultivar Sanilac. The deficiency in phaseolin and lectins is associated with an increased concentration of sulfur amino acids, cysteine and methionine, primarily at the expense of the non-protein amino acid, *S*-methylcysteine, and increased levels of sulfur-rich proteins (Taylor et al., 2008; Marsolais et al., 2010; Yin et al., 2011; Liao et al., 2012). This property is of interest to improve protein quality and relevant to nutritional claims on protein content. The changes in protein composition are associated with increased protein solubility (Taylor et al., 2008).

The objective of the present study was to characterize the genetic polymorphisms responsible for differences in phaseolin and lectin expression between SARC1 and SMARC1N-PN1. To do so, a combination of approaches was used, including re-analysis of a quantitative proteomic dataset coupled with Western blotting and affinity purification, genomic PCR and genomic sequencing. The results identify several polymorphisms associated with storage protein deficiency and shed light on the process of proteome rebalancing in crop seeds.

## Materials and Methods

### Plant Material and Growth

Common bean (*Phaseolus vulgaris* L.) genotypes were grown in a growth cabinet (Environmental Growth Chambers, Chagrin Falls, OH, USA) under 16 h light (300–400 μmol photons m^-2^ s^-1^) and a temperature cycling between 18 and 24°C (Pandurangan et al., 2012). The generation of SARC1, SMARC1-PN1 and SMARC1N-PN1 genetic stocks was described by Osborn et al. (2003). Seeds from parents G12882 and Great Northern 1140 were obtained from the Germplasm Resources Information Network of the United States Department of Agriculture-Agricultural Research Service, Western Regional Plant Introduction Station, Pullman, WS, USA. A number of *Phaseolus coccineus* seeds lacking phaseolin, originally characterized at the CNR in Pisa, Italy (Durante et al., 1989), that are commonly found in local markets in Tuscany, Italy and were kindly provided by Luccarini, were confirmed by examining seed protein profiles for the absence of phaseolin. Mature seed tissue (100 mg) was homogenized in 0.5 × Sample Buffer [4% SDS, 25 mM Tris–HCl pH 6.8, 2.5% (v/v) glycerol] to extract total protein. The extracts were boiled immediately for 5 min, centrifuged for 15 min at room temperature and supernatants were saved. Protein concentration was determined using the Bio-Rad Protein Assay solution (Mississauga, ON, Canada) and bovine serum albumin as standard. Equal amount of protein was separated by SDS-PAGE on a 10% polyacrylamide gel.

### Protein Analysis by Spectral Counting

Quantitative proteomic data (Marsolais et al., 2010) were re-analyzed using Scaffold 2 software (Proteome Software Inc., Portland, OR, USA) against the UniProt database, section *Viridiplantae* (as of March 31, 2009).

### Purification of Mannose Lectin FRIL

Mannose lectin FRIL was purified by affinity chromatography on D-mannose agarose and eluted competitively with methyl α-D-mannopyranoside (Sigma–Aldrich, Oakville, ON, Canada) as described by Colucci et al. (1999). The identity of protein bands was confirmed by LC-MS after tryptic digestion as described in (Marsolais et al., 2010). The peak list was searched against NCBInr/Other green plants using Mascot^1^.

### Analysis of α-Amylase Inhibitor 1 by Western Blot

Mature seed tissue (100 mg) was extracted and protein quantified as described above. Equal amount (2 μg) separated by SDS-PAGE on a 15% polyacrylamide gel was transferred to a nitrocellulose membrane (9 cm × 6 cm) at 15 V for 20 min using a semi-dry transfer apparatus (Bio-Rad Laboratories, Inc.). The membrane was blocked with Odyssey Blocking Buffer (LI-COR Biosciences, Lincoln, NE, USA) at room temperature for 1 h. The membrane was incubated with 1:2000 dilution of anti-α-amylase inhibitor antibodies (Lioi et al., 2007) for 1 h, followed by 1:10,000 dilution of goat IRDye800R Conjugated Affinity Purified Anti-Rabbit IgG (Rockland Immunochemicals Inc., Limerick, PA, USA) for 1 h. Immunodetection was achieved by scanning with an Odyssey Infrared Imaging System (LI-COR). Bands were quantified using ImageStudio ver. 3.1 software (LI-COR).

### DNA Isolation and PCR Genotyping

Leaf tissue was frozen in liquid nitrogen and ground to a fine powder using a mortar and pestle. Genomic DNA was isolated using the GenElute Plant Genomic DNA Miniprep Kit (Sigma–Aldrich) following the manufacturer’s protocol. PCR was carried out using 50 ng of genomic DNA as template for 35 cycles with Taq DNA polymerase and the following gene specific primers: for *ARC1*, F: 5′-AGCAACGACGCCTCCTTCAACG-3′ and R: 5′-CCTTTAAGTTTGGGCCGAGAGCCG-3′; for *arc3-II*, F: 5′-ACTAGCTTCCACCAAGGCGATCC-3′ and R: 5′-TTCTGTCATAGCGGAGGGTGTAGC-3′; for *arc4-I*, F: 5′-AGTATCCGCCCATACAGTAACAATG-3′ and R: 5′-CACGCTGCTGGTAGAGAAGTTG-3′; for *pha-E*, F: 5′-CGCACACACTTGCAACATCCC-3′ and R: 5′-GGTTTGGGGTCCCAGTGAACGT-3′; for α-amylase inhibitor 1, F: 5′-GAAACCTCCTTCAACATCGATGG-3′ and R: 5′-CCCTCACCCAGTCGTAAACTTCT-3′; and for mannose lectin FRIL, F: 5′-GTGGAGGAAACCCTGTGGGTGC-3′ and R: 5′-CGGCTCCTTCACCTCGTTGTTCT-3′. The following primers were used to confirm the presence of *pdlec1* in SMARC1N-PN1 and Great Northern 1140: PhaL-F165, 5′-CTCCTCTTCTCACTATGACAC-3′ and PhaL-R838, 5′-GACTCCAAACTCCACCTTCC-3′. The following primers were used to amplify the β-phaseolin promoter: pBetaPhsF, 5′-CCTTTCTTGGTATGTAAGTCCG-3′ and pAlphaBetaPhsR, 5′-AGTAGAGTAGTATTGAATATGAGTTG-3′. PCR products were sequenced using a 3130XL Genetic Analyzer (Life Technologies, Burlington, ON, Canada).

### Next Generation Sequencing

DNA was isolated using a Qiagen DNeasy Plant Mini Kit (Toronto, ON, Canada). Care was taken to isolate intact genomic DNA. To minimize shearing, the samples were not vortexed and wide-bore pipette tips were used for handling. After the final wash, the samples were eluted in 100 μl of 10 mM Tris-HCl pH 8.0. DNA samples were visualized on a 1% agarose gel. DNA concentration and purity was determined using a Nanodrop 1000 (Thermo Scientific, Wilmington, DE, USA). Genomic DNA samples from Sanilac, SARC1, SMARC1-PN1 and SMARC1N-PN1 were submitted for paired-end read sequencing on an Illumina HiSeq 2000/2500 platform (San Diego, CA, USA) at the Clinical Genomics Centre, Toronto, ON, Canada, following recommended guidelines. Low quality reads were filtered out, resulting in approximately 925–1025 million reads per sample. In addition to paired-end read sequencing, three mate-pair libraries were prepared, short (3.5–4.5 kb), medium (5–7 kb), and large (8–11 kb) using Illumina’s protocol to obtain 50 base pair reads. Samples from each size were multiplexed and run on a single lane. Sequencing data can be found in the short read archive at the National Center for Biotechnology Information, with the following accession numbers: for Sanilac, SRP055506; for SARC1, SRP055509; for SMARC1N-PN1, SRP055510; and for SMARC1-PN1, SRP055511.

Paired-end reads were aligned to the *P. vulgaris* G19833 genome sequence (Schmutz et al., 2014), scaffold assemblies of the BAT-93 genome (Vlasova et al., 2016) and of the OAC-Rex genome (Perry et al., unpublished results^2^), a BAC clone for the APA locus of an arcelin-5 genotype (Kami et al., 2006) and α-phaseolin gene sequences from Sanilac (Anthony et al., 1990; Diniz et al., 2014), as described in O’Rourke et al. (2013). Reads were aligned with BWA using default parameters (Li and Durbin, 2009). Sequence Alignment/Map (SAM) files generated were converted to sorted indexed Binary Alignment/Map (BAM) files using SAMtools (Li et al., 2009). Alignments were visualized with IGV (Robinson et al., 2011).

To assemble paired-end and mate-pair read data, sequencing reads were analyzed by performing FastQC^3^ to get the read profile and evaluate possible sequence contamination. A modified blosum filter was applied to remove duplicate reads using pybloomfaster^4^. Low quality reads were removed using fastq_quality_filter ^5^. A custom adapter trimmer was performed to remove any contaminating sequence identified by FastQC. Reads were reorganized by title for the synchronizer using a custom sort script. A custom synchronizer script was run to ensure that the R1 and R2 files contained the same reads in the same order after filtering. Orphans were saved, but not used. Trimmed files were archived. Paired end reads were assembled into contigs using Ray (Boisvert et al., 2010). A custom contig fractionator script was run to generate a set of artificial paired end reads with controlled overlap for use in the scaffolding. Scaffolding was performed using ALLPATHS-LG (Gnerre et al., 2011). Assembly stats were cross checked with assemblathon_stats.pl. CEGMA was used to identify 248 Core Eukaryotic Genes (CEGs) as an indirect measure of functional completeness of the assembly (Parra et al., 2009).

### Promoter Analysis

Proximal promoter sequences were analyzed and compared using a database of plant *cis*-acting regulatory DNA elements, PLACE ^6^ (Higo et al., 1999).

### Accession Numbers

Additional nucleotide sequence data from this article have been deposited in the GenBank database under accession numbers: [GenBank ID: KU258848] for mannose lectin FRIL from SMARC1N-PN1; [GenBank ID: KU258849] from G12882; [GenBank ID: KU258850] from *P. coccineus*; and [GenBank ID: KU258846] for α-amylase inhibitor like protein from SARC1; [GenBank ID: KU258847] from SMARC1-PN1; and [GenBank ID: KU258845] from SMARC1N-PN1.

## Results

### Proteomic Analysis of Phaseolin and Lectin Composition in SARC1 and SMARC1N-PN1

To understand the effect of storage protein deficiency on the composition of phaseolins and lectins, shotgun proteomic data from total protein extracts from SARC1 and SMARC1N-PN1 (Marsolais et al., 2010) was re-analyzed and quantified with SCAFFOLD software. The results are presented in **Table 1**. SCAFFOLD is particularly adept at assigning spectra to a given accession among a group of closely related proteins, although in all cases, the algorithm reported protein grouping ambiguity except for arcelin-like protein 4 and α-amylase inhibitor like protein. The results confirmed the absence of α-phaseolin and the residual levels of β-phaseolin (Phaseolin precursor, encoded by *Phs*) present in SMARC1N-PN1. In prior analyses, the presence of β-phaseolin in SMARC1N-PN1 had been inferred from the results of two-dimensional gel electrophoresis based proteomics (Marsolais et al., 2010). For lectins, the present results suggest that there are three distinct arcelins as well as arcelin-like protein 4 in SARC1. This new analysis confirms the absence of lectins encoded by *lec4-B17* and *pha-E* in SMARC1N-PN1. Partial compensation by a leucoagglutinating phytohemagglutinin, encoded by *PDLEC2* (Voelker et al., 1986), α-amylase inhibitor like protein, α-amylase inhibitor 1 and mannose lectin FRIL are also apparent in these data.

**Table 1:** Differentially expressed phaseolins and lectins in mature seeds of SARC1 and SMARC1N-PN1 quantified by spectral counting, as unweighted spectrum count, with a minimum of 1 peptide identified with 95% probability (average ± standard deviation); *n* = 3; n.s., not significant.

Protein	Gene name	UniProt accession	SARC1	SMARC1N-PN1	*t*-test *p*-value
Phaseolin precursor	*Phs*	Q43632	826 ± 175	40 ± 34	0.002
α-Phaseolin		Q41115	799 ± 152	0	0.0008
Phaseolin, α-type		P07219	713 ± 122	0	0.0005
Arcelin-1 precursor	*ARC1*	P19329	955 ± 273	0	0.004
Arcelin	*arc3-II*	Q8RVY3	67 ± 11	0	0.0004
Arcelin-like protein 4	*arl4*	Q8RVX7	90 ± 8	0	0.00004
Arcelin	*arc4-I*	Q8RVX4	73 ± 3	0	0.000003
Lectin precursor	*lec4-B17*	Q8RVX5	27 ± 11	0	0.015
Phytohemagglutinin	*pha-E*	Q8RVX6	83 ± 41	0	0.03
Phytohemagglutinin	*pha-L*	Q8RVH2	72 ± 59	7 ± 1	n.s.
Leucoagglutinating phytohemagglutinin	*PDLEC2*	P15231	41 ± 28	121 ± 38	0.04
α-Amylase inhibitor like protein		Q9SMH0	3 ± 1	150 ± 11	0.00002
α-Amylase inhibitor 1		A0T2V3	17 ± 17	311 ± 32	0.0001
Mannose lectin FRIL		Q9M7M4	3 ± 2	116 ± 16	0.0002

### Mannose Lectin FRIL and α-Amylase Inhibitor 1 Are Uniquely Present in SMARC1N-PN1

**FIGURE 1:**
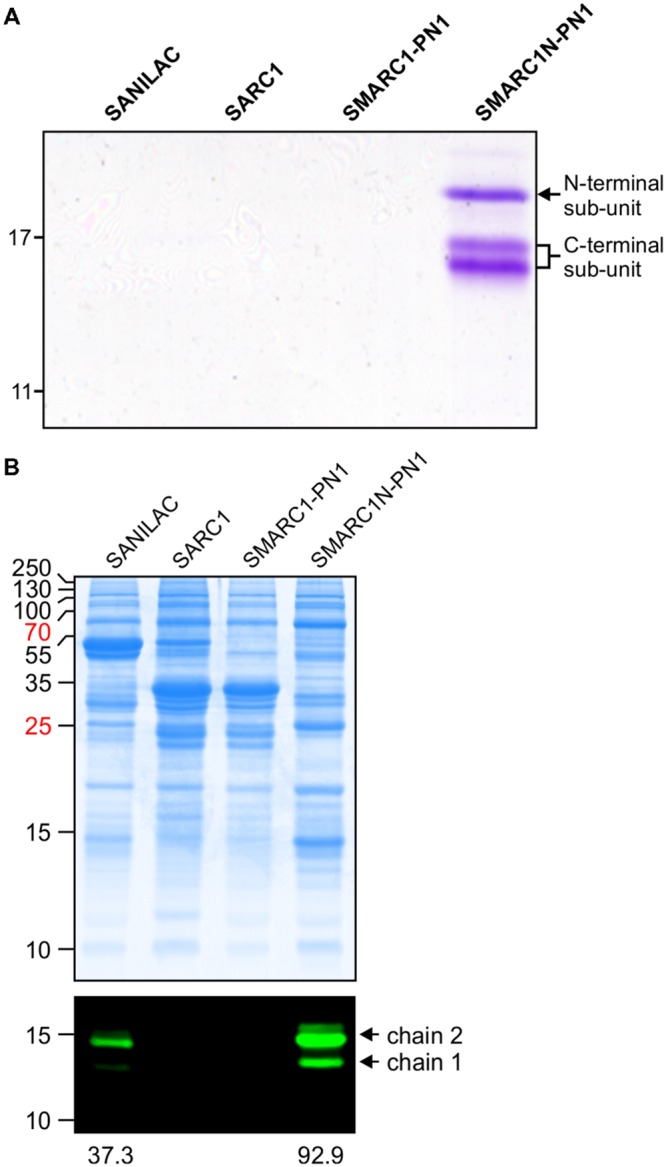
**(A)** Affinity purification of mannose lectin FRIL. SDS-PAGE of mature seed extracts after purification on mannose agarose. The band of 20 kDa corresponds to the N-terminal subunit of mannose lectin FRIL. The bands of 17 and 16 kDa correspond to the C-terminal subunit. The identity of protein bands was confirmed by proteomics (see **Table 2**). **(B)** Immunoblotting of total protein extracts from mature seed with polyclonal antibodies raised against recombinant α-amylase inhibitor. Bands of 14.5 and 13 kDa correspond to chain 2 and chain 1 of α-amylase inhibitor 1, respectively.

For lectins detected at relatively low levels by spectral counting, it was not clear whether they are truly present or whether they are detected based on their high sequence similarity with other lectins. This was further investigated for mannose lectin FRIL and α-amylase inhibitor 1. Mannose lectin FRIL was affinity purified from mature seed of Sanilac, SARC1, SMARC1-PN1 and SMARC1N-PN1 on mannose-agarose and the purified protein analyzed by SDS-PAGE. Protein bands corresponding to mannose lectin FRIL were uniquely present in SMARC1N-PN1 (**Figure 1A**). Three bands were observed having apparent molecular masses of 20, 17, and 16 kDa, respectively. The first one constitutes the N-terminal subunit and the other two the C-terminal subunit (Moore et al., 2000). This was confirmed by a proteomics approach, based on the coverage of each subunit by identified tryptic peptides (**Table 2**).

**Table 2:** Identification of protein bands from SMARC1N-PN1 in **Figure 1A** by LC-MS-MS and Mascot search following trypsin digestion.

Protein band apparent molecular mass (kDa)	Name	GI number	Score	Matches	Number of peptides matching N-terminal	subunit	Number of peptides matching C-terminal subunit	Predicted mass of respective subunit (Da)	Coverage of respective subunit (%)
20	Mannose lectin FRIL	6822274	284	35	35	0	14242.7	33
17	Hypothetical protein	593675212	520	22	2	20	14992.7	74
16	Mannose lectin FRIL	6822274	566	34	4	30	16877.8	41

*The accession named hypothetical protein represents mannose lectin FRIL in the reference G19833 genome (Phytozome accession number PHAVU_007G070100g).*

α-Amylase inhibitor 1 was immunodetected in mature seed protein extracts of Sanilac, SARC1, SMARC1-PN1 and SMARC1N-PN1 using polyclonal antibodies raised against recombinant α-amylase inhibitor. Two major bands of approximately 14.5 and 13 kDa were detected (**Figure 1B**), corresponding to chain 2 and chain 1 of α-amylase inhibitor 1, respectively (Moreno and Chrispeels, 1989; Yamaguchi, 1991). No signal was detected in SARC1 and SMARC1-PN1. Protein levels were higher in SMARC1N-PN1 than in Sanilac, as determined by quantification of the main protein band corresponding to chain 2, by approximately 2.5-fold.

### Analysis and Validation of Lectin Gene Composition by Genomic PCR

**FIGURE 2:**
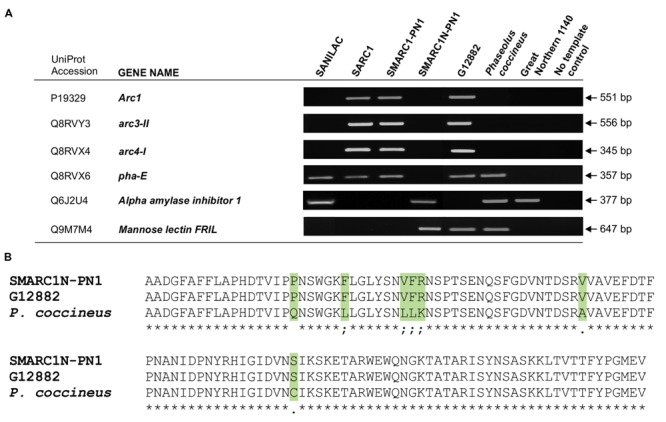
**(A)** Analysis of lectin gene composition by genomic PCR. The presence or absence of lectin genes was evaluated using gene-specific primers. The phaseolin-deficient *P. coccineus* accession is distinct from the one used by Osborn et al. (2003). **(B)** Sequence alignment of conceptual translations of PCR products coding for mannose lectin FRIL.

Based on the above results, analysis of lectin gene composition was conducted by genomic PCR using primers complementary to the coding sequence or, where possible, to the 5′-untranslated region. Samples included the three genetically related lines as well as the parental background Sanilac, the two other parents, G12882 and Great Northern 1140 and a *P. coccineus* phaseolin deficient genotype, supposed to bear the same *phs* null allele of the SMARC1N-PN1 line. The genomic PCR results confirmed the presence of three different arcelin genes in SARC1, SMARC1-PN1, and G12882, the source of arcelin genes in the two lines (**Figure 2A**). In addition, no amplification of *pha-E*, encoding erythroagglutinating phytohemagglutinin, was observed in SMARC1N-PN1 and in Great Northern 1140, the source of lectin deficiency. The genomic PCR data confirmed the presence of the α-amylase inhibitor 1 gene in SMARC1N-PN1 and its absence in SARC1, SMARC1-PN1 and G12882. The α-amylase inhibitor 1 gene was also detected in Sanilac. Mannose lectin is encoded on chromosome 7 and not in the APA locus which is situated chromosome 4. The mannose lectin gene was found to be present in SMARC1N-PN1, G12882 and the *P. coccineus* genotype. Alignment of conceptual translations of PCR products indicated that mannose lectin FRIL originates from G12882 in SMARC1N-PN1, and was likely lost during crossing and propagation of the lines that led to SARC1 and SMARC1-PN1 (**Figure 2B**).

### Genome Sequencing

To gain more insight into the polymorphisms associated with storage protein deficiency, the genomes of the three genetically related lines, SARC1, SMARC1-PN1 and SMARC1N-PN1 and of the recurrent parent, Sanilac were sequenced using a whole-genome shotgun sequencing approach which combined Illumina sequenced fragment libraries to obtain 100 bp paired end reads along with mate-pair libraries of fragments of three different lengths to assist *de novo* assembly, with a sequence read coverage of the estimated genome size greater than 150-fold (Supplementary Table S1). Two different approaches were used to analyze the data. In the first approach, paired end reads were mapped to a reference sequence using Burrows–Wheeler Aligner software. In the second approach, scaffold assemblies of the four genomes were generated using ALLPATHS-LG and analyzed for the genes of interest (Supplementary Table S2).

#### Absence of *lec4-B17* and *pha-E* and Presence of the Pseudogene *pdlec1* in SMARC1N-PN1

**FIGURE 3:**
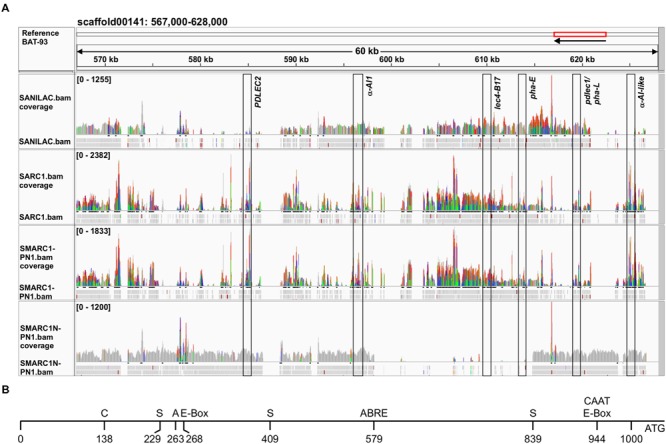
**(A)** Sequence reads of lectin locus aligned to the reference BAT-93 genome. Paired end reads from the four genotypes sequenced by Illumina HiSeq2000/2500 were aligned and visualized with IGV. The positions of the lectin genes are indicated by boxes. Gray indicates sequence identity. Color highlights variant bases compared to the reference sequence; Green for A; Red for T; Orange for G; and Blue for C. *PDLEC2* and α-amylase inhibitor like protein gene (*α-AI-like*) are not covered in Sanilac; likewise α-amylase inhibitor 1 (*α-AI1*) in SARC1 and SMARC1-PN1. BAT-93 and SMARC1N-PN1 have the *pdlec1* allele in place of *pha-L*. **(B)** Schematic view of polymorphisms in the promoter of α-amylase inhibitor like protein between SMARC1N-PN1 versus SARC1 and SMARC1-PN1 giving rise to unique *cis*-regulatory elements in SMARC1N-PN1. Analysis of *cis*-regulatory elements was performed using the Place database. C, CAAACAC element; S, soybean embryo factor 4 binding motif; A, core AACA motif; ABRE, abscisic acid related element.

For analysis of the APA locus, BAT-93, a Mesoamerican genotype, was most similar to SMARC1N-PN1. **Figure 3A** shows the alignment of the paired end reads to the part of the BAT-93 scaffold00141 containing the APA locus, visualized with IGV. Sequences in gray are identical. Color highlights variant bases. Peak height indicates the number of reads aligned. BAT-93 and other genomic templates were annotated manually after blastn against NCBInr and blastx of individual APA coding sequences against UniProt, based on highest sequence identity to a known lectin accession. In order to annotate the genes in the alignments, reads were joined manually to generate a coding sequence which was used for blastx against UniProt. The gene order was found to be conserved across reference genotypes (BAT-93, G19833, OAC-Rex and the BAC-71F18 from the arcelin-5 genotype). However, the composition of APA genes varied. For the phytohemagglutinin gene located between *pha-E* and the α-amylase inhibitor like protein gene (**Figure 3A**), different alleles were found to be present. G19833 and OAC-Rex have *pha-L*, as do Sanilac, SARC1 and SMARC1-PN1. BAT93 and SMARC1N-PN1 have the *pdlec1* pseudogene, previously characterized from Pinto UI111 (Voelker et al., 1986). The presence of the *pdlec1* pseudogene in SMARC1N-PN1 and in Great Northern 1140 was confirmed by PCR amplification and sequencing of the PCR products. The sequences isolated were 100% identical to that reported by Voelker et al. (1986). The *pdlec1* allele is characterized by a deletion of a single nucleotide, cytosine, after position 32 of the coding sequence, resulting in a premature stop codon at position 132. BAT93 and SMARC1N-PN1 also share the *PDLEC2* gene, coding for a leucoagglutinating phytohemagglutinin isoform, further extending the homology with Pinto UI111 (Voelker et al., 1986). G02771, a wild, arcelin-5 genotype, has the arcelin-5 phytohemagglutinin (Kami et al., 2006). Arcelin-5 phytohemagglutinin is 99% identical to *pdlec1*, but is not a pseudogene. The alignment in **Figure 3A** confirmed the absence of *lec4-B17* and *pha-E* in SMARC1N-PN1. The alignment also suggested the absence of *PDLEC2* and α-amylase inhibitor like protein gene in Sanilac. *PDLEC2* and the α-amylase inhibitor 1 gene appeared only partially covered in SARC1 and SMARC1-PN1 suggesting their absence in these genotypes. This conclusion is supported by the Western blotting and genomic PCR data for α-amylase inhibitor 1 (**Table 1, Figures 1** and **2**). It was not possible to verify the presence of *PDLEC2* by genomic PCR due to high degree of sequence identity between leucoagglutinating phytohemagglutinin genes.

#### Multiple Polymorphisms in the Promoter of α-Amylase Inhibitor Like Protein Are Associated with Increased Expression in SMARC1N-PN1

The scaffold assemblies of SARC1, SMARC1-PN1 and SMARC1N-PN1 contained a full length coding sequence for α-amylase inhibitor like protein (Supplementary Table S3). This is consistent with alignments of paired end reads (**Figure 3A**). Promoter sequences were aligned. Polymorphic sites were searched for differences in *cis* regulatory motifs between SMARC1N-PN1 versus SARC1 and SMARC1-PN1 using the PLACE database (Higo et al., 1999). This analysis revealed the presence of multiple individual positive *cis*-regulatory motifs that are unique to SMARC1N-PN1 (**Figure 3B**). These include a CAAACAC element characterized in the napin promoter of *Brassica napus* (Stålberg et al., 1996), three instances of the soybean embryo factor 4 binding motif characterized by Lessard et al. (1991), a core AACA motif (5′-AACAAAC-3′) present in the rice glutelin promoter (Wu et al., 2000), and an abscisic acid related element (5′-ACGTGGC-3′) required for *RD29B* expression in *Arabidopsis* seed (Nakashima et al., 2006). There are also two instances of the E-box (Stålberg et al., 1996), the second overlapping with a CAAT box, proximal to the start codon (Shirsat et al., 1989).

#### Differences in β-Phaseolin Accumulation Correlate with a Single Nucleotide Polymorphism Converting a G-Box Motif into an ACGT Motif in the Promoter of SMARC1N-PN1

**FIGURE 4:**
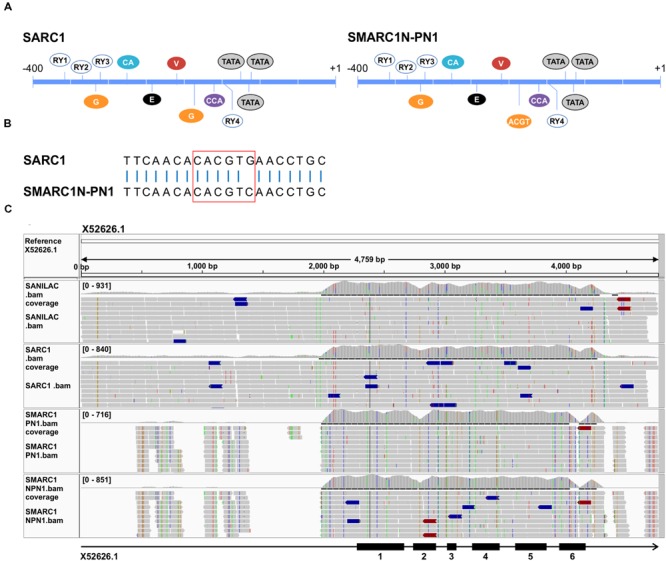
**(A)** Schematic view of *cis*-regulatory elements present in the proximal promoter of β-phaseolin in SARC1 and Sanilac versus SMARC1N-PN1 and SMARC1-PN1. Regulatory motifs are designated as in Chandrasekharan et al. (2003). A single nucleotide polymorphism converts a G-box in SARC1 and Sanilac to an ACGT motif in SMARC1N-PN1 and SMARC1-PN1 **(B)**. G, G-box; E, E-box; C, CACA box; V, vicillin box; CCA, CCAAT box. **(C)** Sequence read alignments to the α-phaseolin gene from Sanilac (accession number X52626). The bar below shows the intron-exon structure with exons numbered as black boxes. The alignment shows incomplete coverage of the promoter region in SMARC1-PN1 and SMARC1N-PN1.

Several functional regions within the β-phaseolin promoter have been defined by deletion analyses (Bustos et al., 1991; van der Geest and Hall, 1996; Chandrasekharan et al., 2003). These include four RY repeat motifs (5′-CATGC/TA-3′) (Bobb et al., 1997), a G-box binding motif (5′-CACGTG-3′) and E-box motif (5′-CACCTG-3′) (Kawagoe et al., 1994), CACA element (Li et al., 1999), vicillin box (Chern et al., 1996a,b), ACGT motif and CAAT box (5′-CCAAAT-3′ in *Phs* promoter) (Li et al., 1999) (**Figure 4A**). Deletion analysis studies previously showed that the G-box, RY motifs, E-box and CAAT box are required for high level expression of a reporter in transgenic *Arabidopsis* seed (Chandrasekharan et al., 2003). Binding of the B3-domain containing VP1/ABI3 member PvAlf is required for β-phaseolin expression (Bobb et al., 1995). Gene activation is a two-step process, requiring PvAlf and abscisic acid (Li et al., 1999). Each of these two steps is associated with specific chromatin modifications (Ng et al., 2006) resulting in nucleosomal displacement over the three phased TATA boxes (Li et al., 1998). PvAlf binds to the promoter via the RY repeat motifs (Carranco et al., 2004). ABI5, a bZIP transcription factor, acts downstream from abscisic acid in β-phaseolin expression (Ng and Hall, 2008). Deletion analysis results implicated the G-box as the major abscisic acid responsive element in the β-phaseolin promoter.

In SMARC1N-PN1, β-phaseolin accumulates at lower levels than in SARC1, by approximately 20-fold (**Table 1**). Analysis of paired end read alignments to the reference genome G19833 revealed complete coverage of the β-phaseolin gene in the four genotypes. Focusing on the proximal promoter, all of the *cis*-regulatory elements described above except one were conserved (**Figures 4A,B**). Sanilac and SARC1 have a second G-box downstream from the first element. A single nucleotide polymorphism converts the ACGT motif present in SMARC1-PN1 and SMARC1N-PN1 into this second G-box motif (**Figures 4A,B**). This polymorphism was confirmed by genomic PCR and sequencing of the PCR products. In addition, the same fragment was amplified from phaseolin-containing and phaseolin-deficient *P. coccineus* genotypes. Both had the ACGT motif present. These results suggest that the single nucleotide polymorphism was introduced from *P. coccineus* into SMARC1-PN1 and SMARC1N-PN1, abrogating the second G-box motif. The present study associates this single nucleotide polymorphism with the genotypic difference in β-phaseolin accumulation.

For α-phaseolin, read alignments to reference sequences from Sanilac (Anthony et al., 1990; Diniz et al., 2014) showed a complete coverage of the coding section of the gene in all four genotypes (**Figure 4C**). Surprisingly, some polymorphisms were observed between the alignment of reads from Sanilac and the reference sequences. Polymorphisms in phaseolin exons and introns clustered in pairs between Sanilac/SARC1 and SMARC1-PN1/SMARC1N-PN1, as expected. The presence of polymorphisms in SMARC1-PN1/SMARC1N-PN1 did not introduce premature stop codons, or affect intron splicing as predicted by GeneSeqer (Usuka et al., 2000). Promoter sequences were poorly covered in the alignment with SMARC1-PN1/SMARC1N-PN1, with large gaps upstream of the proximal promoter. Although scaffold assemblies contained sequences having similarity to phaseolin in Sanilac and SARC1 (Supplementary Table S4), these sequences were too fragmentary to reach a definitive conclusion on the nature of the polymorphism(s) responsible for the absence of α-phaseolin accumulation in SMARC1N-PN1.

#### Differences in Legumin Accumulation Are Not Associated with Genetic Polymorphisms

In SMARC1N-PN1, the most abundant protein in mature seed is the 11S globulin legumin (Marsolais et al., 2010). Blastx search of scaffold assemblies with the conceptual translation of legumin (Yin et al., 2011) identified one major scaffold per genotype. Sequences were extracted from the scaffold 247 for SARC1 and scaffold 972 for SMARC1N-PN1 and aligned. The alignment revealed the absence of polymorphism between the proximal 670 bp promoter sequences from the two genotypes (data not shown).

## Discussion

The goal of this study was to identify genetic polymorphisms associated with storage protein deficiency and proteome rebalancing in common bean, using the genetically related lines SARC1, SMARC1-PN1 and SMARC1N-PN1 and their parental background Sanilac. The three lines are genetic stocks exhibiting a similar percentage of the Sanilac background (83.6–87.5) (Osborn et al., 2003). They are expected to contain significant genetic variability coming from other parents, which include G12882, *Phaseolus coccineus* and Great Northern 1140. The re-analysis of proteomic data confirmed the identity of phaseolin and lectin isoforms which are affected by, or compensate for, seed storage protein deficiency. Arcelin genotypes are classified into types which are generally considered to contain a specific arcelin allele (Osborn et al., 1986; Lioi et al., 2003). Although arcelin-1 was the major arcelin quantified in SARC1, the proteomic and PCR genotyping data confirmed the presence of two other arcelin genes beside *Arc1, arc3-II* and *arc4-I*. Hartweck et al. (1991) had previously noted the presence of different arcelin variants in SARC1, differing in subunit composition (dimer vs. tetramer) and N-terminal sequence. According to the results of read alignments and genomic PCR, the deficiency in erythroagglutinating phytohemagglutinin and lectin appears due to the absence of the corresponding genes, *pha-E* and *lec4-B17*, respectively, in SMARC1N-PN1. SMARC1N-PN1 integrates a distinct allele substituting for *pha-L*, the pseudogene *pdlec1*, and *PDLEC2*. These had been identified from another genetic source of lectin deficiency, Pinto UI111 (Voelker et al., 1986). The present results suggest that Great Northern 1140 and Pinto UI111 share the same APA locus (Osborn and Bliss, 1985). These two genotypes are representative of market classes belonging both to the Durango land race, derived from the Middle American center of domestication (Singh et al., 1991; Mensack et al., 2010). While the genetic relationship between these two genotypes is unknown, the results suggest that they share a common origin (McClean and Myers, 1990). The reference genotype BAT-93, also a Middle American genotype, shares the *pdlec1* allele and *PDLEC2*, although it contains functional copies of *pha-E* and *lec4-B17*.

The deficiency in α-phaseolin is likely to be due to the partial or complete absence of the gene in SMARC1-PN1 and SMARC1N-PN1. Notably, the promoter sequence was poorly covered in read alignments. The high degree of sequence identity between phaseolin genes precluded the design of primers specific to α-phaseolin. The quality of the *de novo* genome assemblies was insufficient to reach a definitive conclusion regarding this gene. The high degree of sequence identity between phaseolin or lectin coding sequences hampers the assembly process. In future, the addition of long reads may facilitate gap closing and scaffold joining in the assemblies. This may help to clarify the status of the α-phaseolin gene in SMARC1-PN1 and SMARC1N-PN1. The large decrease in β-phaseolin accumulation in SMARC1N-PN1 as compared with SARC1, of approximately 20-fold, was associated with a single nucleotide polymorphism converting a G-box motif into an ACGT motif in the proximal promoter. The originally characterized sequence from Tendergreen (Slightom et al., 1983), an Andean genotype, as well as the reference Andean genome G19833, have the ACGT motif like SMARC1-PN1 and SMARC1N-PN1. This ACGT motif was shown to have little influence on the levels of reporter gene expression in transgenic *Arabidopsis* seeds (Chandrasekharan et al., 2003). However, an upstream G-box motif was required for high level expression. This single nucleotide polymorphism was likely introgressed from the *P. coccineus* accession, as it was present in *P. coccineus* genotypes examined in this study.

The results of this study also shed light on the mechanisms leading to compensation by residual lectins. Mannose lectin FRIL and α-amylase inhibitor 1 genes are absent from SARC1 and present in SMARC1N-PN1. The levels of α-amylase inhibitor 1 are slightly higher in SMARC1N-PN1 than in Sanilac, by 2.5-fold, the gene being present in a different genomic context. α-Amylase inhibitor-like protein is of particular interest. Unlike β-phaseolin, the large difference in protein accumulation, of approximately 50-fold, is associated with multiple polymorphisms in the proximal promoter, introducing eight potential positive *cis*-regulatory elements related to seed expression specific to SMARC1N-PN1, including a CAAT box in the right location, important for high level expression of tissue-specific genes. For legumin, the major storage protein in SMARC1N-PN1, accounting for close to 20% of total protein, the proximal promoters of 670 bp in size were identical between SARC1 and SMARC1N-PN1. While legumin levels are raised by close to 7-fold in SMARC1N-PN1 relative to SARC1, its transcripts levels were elevated by approximately 2-fold during seed development (Liao et al., 2012). These results are consistent with those obtained with soybeans in which expression of major seed storage proteins was down-regulated by RNAi (Schmidt et al., 2011). The authors concluded that while seed protein concentration appears to be genetically determined, the identity of proteins compensating for storage protein deficiency in these lines is determined at the post-transcriptional level, in the absence of genetic polymorphisms (Herman, 2014). The identification of variants at the phaseolin and APA loci in the present study may also be useful for genetic diversity analyses and marker-assisted breeding in common bean.

## Author Contributions

SP, FY, BC, SH, WC, KP, and FM designed the research; SP, MD, FY, SM, FM analyzed data; GP contributed data; FS and RB contributed germplasm and reagents; all authors contributed to writing of the manuscript.

## Conflict of Interest Statement

The authors declare that the research was conducted in the absence of any commercial or financial relationships that could be construed as a potential conflict of interest.

## Footnotes

1 http://www.matrixscience.com

2 http://www.beangenomics.ca

3 http://www.bioinformatics.babraham.ac.uk/projects/fastqc/

4 https://github.com/brentp/pybloomfaster

5 http://hannonlab.cshl.edu/fastx_toolkit/

6 https://sogo.dna.affrc.go.jp/cgi-bin/sogo.cgi?sid=&lang=en&pj=640&action=page&page=newplace
