# Supplementary material for: Genomic Analysis of Storage Protein Deficiency in Genetically Related Lines of Common Bean (Phaseolus vulgaris)
Source: Front Plant Sci. 2016 Mar 31;7:389. doi: 10.3389/fpls.2016.00389 (PMC4814446; doi:10.3389/fpls.2016.00389)
Supplement: Supplementary file 1 [file Table_S1-S4.DOCX]

Supplementary Material

Genomic analysis of storage protein deficiency in common bean (*Phaseolus vulgaris*)

**Sudhakar Pandurangan, Marwan Diapari, Fuqiang Yin, Seth Munholland, Gregory Perry, B. Patrick Chapman, Shangzhi Huang, Francesca Sparvoli, Roberto Bollini, William Crosby, K. Peter Pauls and Frédéric Marsolais^*^**

*** Correspondence:** Corresponding Author: [Frederic.Marsolais@agr.gc.ca](mailto:Frederic.Marsolais@agr.gc.ca)

**Supplementary Table S1 | Summary of sequencing information**

| **Genotype** | **Library** | **Total reads** | **Total bases (Mbases)** | **Sequencing depth** |
| --- | --- | --- | --- | --- |
| Sanilac | Paired-end | 925,794,123 | 92,503 | 158 |
|  | Mate-pair 3.5-4.5 kb | 114,711,596 | 5,850 | 10 |
|  | Mate-pair 5-7 kb | 95,091,810 | 4,849 | 8 |
|  | Mate-pair 8-11 kb | 113,860,024 | 5,807 | 10 |
| SARC1 | Paired-end | 1,013,697,395 | 101,262 | 172 |
|  | Mate-pair 3.5-4.5 kb | 107,665,812 | 5,491 | 9 |
|  | Mate-pair 5-7 kb | 124,209,624 | 6,335 | 11 |
|  | Mate-pair 8-11 kb | 96,040,264 | 4,899 | 8 |
| SMARC1-PN1 | Paired-end | 962,447,059 | 96,116 | 164 |
|  | Mate-pair 3.5-4.5 kb | 142,569,650 | 7,271 | 12 |
|  | Mate-pair 5-7 kb | 111,520,870 | 5,688 | 10 |
|  | Mate-pair 8-11 kb | 144,586,446 | 7,374 | 13 |
| SMARC1N-PN1 | Paired-end | 943,532,056 | 94,663 | 161 |
|  | Mate-pair 3.5-4.5 kb | 151,020,260 | 7,702 | 13 |
|  | Mate-pair 5-7 kb | 141,672,576 | 7,225 | 12 |
|  | Mate-pair 8-11 kb | 121,213,040 | 6,181 | 11 |

**Supplementary Table S2 | Summary of genome assemblies**

|  | **Sanilac** | **SARC1** | **SMARC1-PN1** | **SMARC1N-PN1** |
| --- | --- | --- | --- | --- |
| Number of contigs | 45,929 | 46,190 | 56,003 | 51,607 |
| Number of scaffolds | 7,723 | 7,804 | 9,016 | 7,743 |
| Total scaffold length, with gaps (bp) | 429,542,446 | 407,675,499 | 403,280,814 | 358,550,216 |
| Total scaffold length as percentage of genome size | 73.2 | 69.4 | 68.7 | 61.1 |
| N50 contig size (bp) | 15,992 | 16,498 | 10,942 | 9,001 |
| N50 scaffold size (kb) | 324,563 | 282,533 | 222,282 | 199,130 |
| GC contents in scaffolds (%) | 26.9 | 27.1 | 26.2 | 25.4 |
| Complete (%) to CEGs by CEGMA pipeline | 85.5 | 84.7 | 85.9 | 85.5 |
| Partial (%) to CEGs by CEGMA pipeline | 96.0 | 94.8 | 96.0 | 94.8 |

**Supplemental Table S3 | Full length coding sequences of lectins encoded at the APA locus in scaffold assemblies.**

| **Genotype** | **Scaffold** | **Subject name** | **Subject ID** | **Query start** | **Query end** | **Subject start** | **Subject end** | **Percent identity** |
| --- | --- | --- | --- | --- | --- | --- | --- | --- |
| Sanilac | 107 | α-Amylase inhibitor 1 | EF087883 | 634990 | 634256 | 1 | 735 | 99 |
| SARC1 | 580 | α-Amylase inhibitor like protein | D49828 | 172608 | 173500 | 1 | 901 | 97 |
|  | 1871 | *arl4* | AJ439619 | 21626 | 22366 | 1 | 723 | 89 |
| SMARC1-PN1 | 1232 | α-Amylase inhibitor like protein | D49828 | 65280 | 66068 | 1 | 789 | 99 |
|  |  | *lec4-B17* | AJ439715 | 80045 | 80860 | 1 | 816 | 99 |
| SMARC1N-PN1 | 47 | α-Amylase inhibitor like protein | D49828 | 58990 | 59890 | 1 | 901 | 99 |
|  |  | α-Amylase inhibitor 1 | AY603476 | 71523 | 72257 | 1 | 735 | 100 |

**Supplemental Table S4 | α- and β-phaseolin sequences in scaffold assemblies. Scaffolds were searched by blastn against Sanilac α-phaseolin gene (NCBI accession X52626) and β-phaseolin gene (J01263).**

| **Genotype** | **Scaffold** | **Range** | **Subject** | **Subject range** | **Strand** | **Percent identity to subject** |
| --- | --- | --- | --- | --- | --- | --- |
| Sanilac | 14 | 26162..28444 | α-Phaseolin | 1..2280 | Plus/minus | 99 |
|  | 14 | 23823..24806 | α-Phaseolin | 3781..4499 | Plus/minus | 99 |
|  | 14 | 24295..24806 | β-Phaseolin | 2992..3502 | Plus/minus | 98 |
|  | 14 | 26162..26456 | β-Phaseolin | 1181..1476 | Plus/minus | 99 |
|  | 14 | 481249..481422 | β-Phaseolin | 11..184 | Plus/minus | 99 |
|  | 14 | 485531..485572 | β-Phaseolin | 1..42 | Plus/minus | 98 |
| SARC1 | 68 | 31352..33610 | α-Phaseolin | 1..2256 | Plus/minus | 99 |
|  | 68 | 29071..29596 | α-Phaseolin | 4239..4764 | Plus/minus | 99 |
|  | 68 | 31352..31622 | β-Phaseolin | 1181..1452 | Plus/minus | 99 |
|  | 68 | 477833..478016 | β-Phaseolin | 1..184 | Plus/minus | 99 |
|  | 68 | 29543..29596 | β-Phaseolin | 3449..3502 | Plus/minus | 100 |
